# Moxifloxacin-warfarin interaction

**DOI:** 10.3402/jchimp.v1i4.11512

**Published:** 2012-01-26

**Authors:** Yan Ji, Youssef Hokayem

**Affiliations:** Department of Medicine, Union Memorial Hospital, Baltimore, MD, USA

**Keywords:** warfarin, moxifloxacin, PT/INR, hypoalbuminemia

## Abstract

Two case reports presented here show elevated prothrombin time/international normalized ratios (PT/INR) following coadministration of warfarin and moxifloxacin. Although the underlying mechanism of this interaction still remains unclear, health care providers should be careful when prescribing moxifloxacin to patients on warfarin therapy, especially to patients with low albumin levels. More frequent monitoring of INR in these patients may be warranted.

Warfarin was approved for use in 1950s and is still widely used due to its effectiveness in multiple medical conditions. It is the most widely prescribed anticoagulant in North America.

The spectrum of its interaction with other commonly used drugs is not yet completely established. Warfarin is mainly metabolized by cytochrome P450. Most of the known drug interactions are with drugs metabolized through the same pathway. Studies have shown that fluoroquinolones (FQ) including ciprofloxacin, levofloxacin, and norfloxacin may enhance warfarin anticoagulation mainly through this mechanism (1, 2). Moxifloxacin does not undergo cytochrome P450 metabolism. For this reason, it was initially accepted as an alternative FQ frequently prescribed to patients on warfarin. However, random cases reported in the past decade indicate that moxifloxacin may increase warfarin anticoagulation effect. Moreover, even though the potential interaction between these two agents has been recognized recently, the underlying mechanism remains unclear.

The two new cases of warfarin-moxifloxacin interactions reported here add to the previously published 12 case reports and highlight the importance of such an interaction (3–6). Potential mechanisms are discussed. We believe that because of the unique metabolism of moxifloxacin, hypoalbuminemic patients, as in our cases, may be particularly at risk.

## Case report

### Case 1

A 53-year-old patient, nursing home resident was admitted to the hospital for abdominal wall and left lower extremity cellulitis with fever (101.7°F) and leukocytosis.

Her medical history included atrial fibrillation for which she had been on warfarin therapy (5 mg daily with therapeutic INR 2–3).

**Fig. 1:**
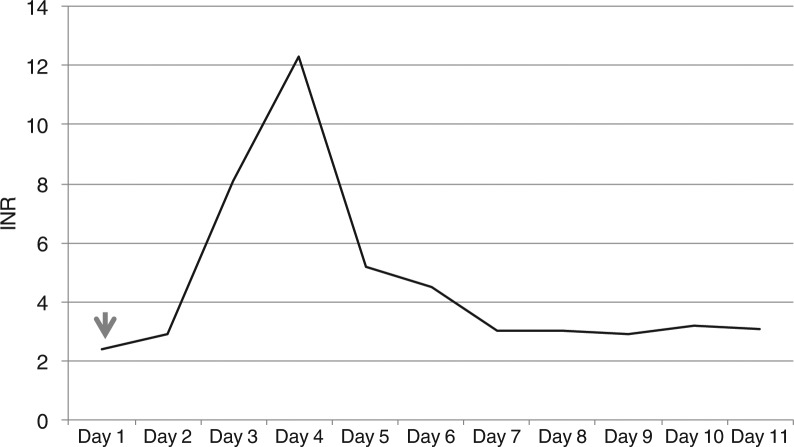
INR curve and timing of moxifloxacin administration in Case 1 (arrow pointing the day that patient received moxifloxacin).

The patient received a seven-day course of ciprofloxacin at the nursing home prior to the admission. Because of an extensive antibiotic allergy profile, the patient was started on clindamycin 600 mg intravenously every eight hours for her cellulitis. She had received one dose of moxifloxacin 400 mg in the ER for suspicion of pneumonia on chest X-ray. The moxifloxacin was discontinued because chest X-ray findings were interpreted as atelectasis, not pneumonia. Warfarin was continued at the same dose. Blood cultures grew 2 out of 2 B hemolytic Group G streptococci. Her laboratory workup on admission showed anemia of chronic disease, normal liver enzymes, albumin of 2.1 g/dL, INR of 2.4, normal electrolyte, and kidney function panels. On day three of hospitalization, the patient's INR became supratherapeutic at 8.1; it peaked at 12 on day four. Warfarin was discontinued and the patient received two doses of vitamin K 5 mg each on day three and day four with adequate INR level correction. The trend of INR level in relation to moxifloxacin administration is illustrated in Fig. 1. Clindamycin was discontinued on day five. No bleeding complications were documented. The hospital course was complicated by altered mental status. However, the patient eventually stabilized and was discharged on day 11 to the nursing home with an INR of 3.1. The plan was to resume warfarin after discharge once the INR becomes therapeutic.

### Case 2

An 80-year-old Caucasian man, nursing home resident with a history of diabetes mellitus, stage III right foot diabetic ulcer status post debridement two months prior to admission on broad-coverage of antibiotics (vancomycin, metronidazole and ceftriaxone), chronic kidney disease, hypertension, and hyperlipidemia, presented to the ER with altered mental status.

On admission, relevant laboratory tests showed: normocytic anemia with Hgb 8.8; slightly elevated Alkaline Phosphatase of 143, Albumin 2.7 g/dL, with otherwise normal liver function tests and acute renal failure with BUN 70, Cr 4.42 (baseline creatinine of 2).

**Fig. 2:**
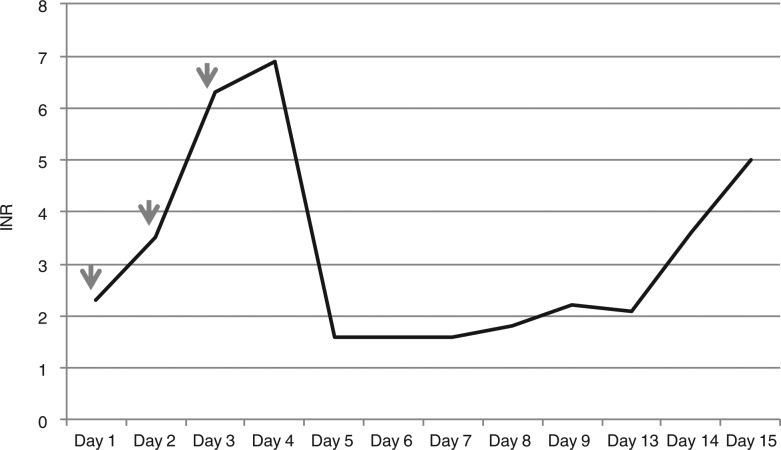
INR curve and timing of moxifloxacin administration in Case 2 (arrows pointing the days that patient received moxifloxacin).

A review of his recent history found that patient had an acute deep vein thrombosis (DVT) in the right subclavian vein, secondary to a peripherally inserted central catheter (PICC) line placed several days prior. The line was removed and he was subsequently started on anticoagulation as an outpatient. Three days after starting warfarin (5mg nightly) treatment, the patient's INR was 2.3. On day one, when he was found to have right lower lobe infiltrates on chest X-ray, patient was started on moxifloxacin for possible pneumonia. His INR (Fig. 2) went up to 3.5, 24 hours after initiation of moxifloxacin (Day two in Fig. 2), 6.3 on Day three and then 6.9 on Day four, when he was admitted to hospital. He received a total of three doses of moxifloxacin.

Because of the supratherapeutic INR, he received two doses of 10mg vitamin K and four units of fresh frozen plasma on Day four and five. His INR decreased to 1.6 subsequently. Warfarin was resumed two days later (Day seven in Figure 2). Unfortunately, his hospital course was complicated by sepsis and multi-organ failure (respiratory, renal and heart). He eventually died despite aggressive medical management. His INR was mostly within the therapeutic range (Day 9 to Day 13 in Fig. 2) till the last two days, when it went up from 2.2 on day 13 to 5 on day 15. This increase was attributed to coagulopathy secondary to uncontrolled sepsis.

## Discussion

Warfarin acts by inhibiting the synthesis of active vitamin K dependent proteins involved in blood coagulation, principally factors II, VII, IX, and X. These factors are synthesized in the liver in precursor form and are activated by carboxylation of specific glutamic acid residues which require vitamin K in its reduced form as a cofactor.

Warfarin has a very narrow therapeutic range, and its anticoagulation effect is frequently altered by various factors, such as drug-drug interactions and patient's medical conditions. Any factor that affects the pharmacodynamics and/or pharmocokinetics of warfarin can cause either subtherapeutic or supratherapeutic INR, and may lead to serious consequences, such as new thromboembolic or bleeding events respectively (7).

The two cases presented here highlight the potential interactions between warfarin and moxifloxacin. In both of our patients, however, other antibiotic agents must be considered: ciprofloxacin and clindamycin in Case 1; metronidazole and ceftriaxone in Case 2. Three of these drugs were chronically used and are unlikely to explain the acute change of INR. In Case 1, the patient received a five-day course of clindamycin after admission. Clindamycin, however, has only been reported in one isolated case (8) to enhance the effect of warfarin by suppression of gut flora and subsequent intrinsic decrease of vitamin K, very unlikely in this patient.

Three potential mechanisms should be considered to explain the interaction between warfarin and moxifloxacin: firstly, FQ cytochrome P450 metabloism effect of fluoroquinolones. However, among the fluoroquinolones, moxifloxacin is uniquely metabolized through glucuronide and sulfate conjugation. Secondly, moxifloxacin depletes vitamin K producing gut flora. We do not believe that this can be the case in our patients, who received one and three doses of moxifloxacin, respectively. Thirdly, moxifloxacin may cause transient elevation of free warfarin level by displacing warfarin from its plasma protein binding site (2, 9). We think that this is most likely the cause. This phenomenon may be more prominent in patients with very low albumin levels as was the case in both of our patients.

Many patients on warfarin therapy are elderly who often have other serious medical problems, such as hepatic or renal diseases and malnutrition, which all can lead to profound hypoalbuminemia. We believe that this may pose a greater risk for this patient population when they are on warfarin treatment. Actually, O'Connor (3) also noticed this phenomenon in a previous case report. All three patients in their report were under-weighted (36–54 Kg) and had moderate to severe decrease of albumin levels (1.8–3.2 g/dL). Their INR was dramatically elevated (7.4, 7.9 and 10 respectively) after starting moxifloxacin treatment (O'Connor, 2003). This is consistent with our observation.

## Conclusion

The substantial increase of INR in our two hypoalbuminemic patients on warfarin therapy after a brief exposure to moxifloxacin is concerning. Close monitoring of the INR level is warranted in this setting to prevent supratherapeutic anticoagulation effect.
